# Potential of galled leaves of Goji (*Lycium chinense*) as functional food

**DOI:** 10.1186/s40795-020-00351-w

**Published:** 2020-07-07

**Authors:** Po-Yen Chen, Tin-Han Shih, Kai-Chieh Chang, Jhin-Syuan Wang, Chi-Ming Yang, Yu-Sen Chang

**Affiliations:** 1 Biodiversity Research Center, Academia Sinica, Nangang, Taipei, 115 Taiwan; 2 Department of Horticulture and Landscape Architecture, National Taiwan University, Daan, 106 Taipei, Taiwan; 3 Miaoli District Agricultural Research and Extension Station, Guannan, Miaoli County, 363 Taiwan

**Keywords:** *Lycium chinense*, Goji, Antioxidant, Chlorogenic acid, Gall, Health food

## Abstract

**Background:**

Goji (*Lycium*) is a popular traditional health food, and its fruit and root extracts have been found to possess antioxidant, anti-inflammatory, and hypocholesterolemia-inducing abilities. Goji leaves also contain high amounts of phenolic compounds, similar to its fruit, and their extracts also exhibit several pharmaceutical effects. The induction of galls on Goji leaves reduces their photosynthetic ability and fruit yield, which raise their farming costs, thereby leading to economic loss. However, the defense mechanisms induced by infection may elevate the secondary metabolite content of the leaves, which might provide more nutritive compounds.

**Method:**

Content of chlorophyll, carotenoids, polyphenols, and flavonoids in the extracts of normal and infected Goji leaves (*L. chinense*) were analyzed. The relative content of chlorogenic acid and rutin, two major phenolic compounds in Goji leaves, were determined by LC-MS/MS. Antioxidant activity was presented by demonstrating the DPPH scavenging percentage. The extract of Goji fruit (*L. barbarum*) was also analyzed to show a comparative result.

**Results:**

In this study, we found that in infected Goji leaves, the polyphenol content was significantly increased. The level of chlorogenic acid was increased by 36% in galled leaves. The content of rutin in galled leaves was also elevated. Testing the antioxidant activities also showed that the extracts of galled leaves have higher DPPH scavenging abilities.

**Conclusions:**

Our results demonstrated that galled Goji leaves have higher functional value, and may have potential as being consumed as health food.

## Background

The Goji plant (primarily *Lycium barbarum* and *L. chinense*) has been used for traditional medicine and cuisine in East Asia for centuries, and recently, these uses of Goji plant have been receiving more attention in western countries [1, 2]. The Goji fruit, also known as the wolfberry or Goji berry, is the main product commercialized as health food. Goji berry contains high amount of functional components, including sugars [3], carotenoids [4], and essential fatty acids [5].The polysaccharides extracted from wolfberries contain high antioxidative properties and have the ability to treat and prevent multiple chronic diseases, including cancer, diabetes, atherosclerosis, and male infertility [6–9]. The root bark of Goji (Lycii Radicis Cortex) has been used in treating hypertension and reducing serum glucose and lipids [10–12]. Other functions of the Lycii Radicis Cortex extract include inhibition of CCl_4_-induced hepatic damage and protection of skin from UVB radiation [13, 14].

Goji leaves are herbs that are traditionally used in tea and cuisine and has been recognized as a health food. It has also been found that the extracts of Goji leaves possess multiple pharmacological effects, including antimicrobial, antioxidant, and anti-diabetic effects [15–18]. However, the comprehensive profiles of biochemical compounds in Goji leaves have only been identified in recent decades. Goji leaves contain high amounts of specific flavonoids and phenolic acids, such as chlorogenic acid, quercetin, and rutin [16, 19]. Comparative studies have demonstrated the differences in compound contents between the leaves of *L. babarum* and *L. chinense*, with higher amounts of chlorogenic acid present in the leaves of *L. chinense* [16]. After comparing the compounds in the fruit, leaves, and root barks of Goji, results indicate that Goji leaves are valuable sources for obtaining chlorogenic acid and rutin [18]. Moreover, Goji leaves also contain polysaccharides that exert high superoxide and DPPH scavenging abilities, therefore having high antioxidative activity.

Goji plants are susceptible to the Goji gall mite, *Aceria kuko* [20], which is a pest that induces yellow-green, bead-like galls in the gall sector of the leaves. Severe infection causes the loss of photosynthetic ability and eventually reduces fruit production, and the infected leaves are regarded as waste. Pesticides are often used in controlling gall mite-induced damage; however, the application of pesticides is dependent on environmental temperatures and on the growing season in order to maximize effectiveness, yet its impact is still limited [21]. Pesticide residue is also one of the concerns when using chemicals on leaves. Although defoliation of galled leaves is relatively effective in practice, this method is costly and time consuming [22].

The infection of galls has been found to induce the biosynthesis of bioactive ingredients, such as flavonoids and phenolic acids, in both plant and gall tissues [23–25], thus, it might be a good idea to take advantage of the gall infection. An excellent example of benefiting from gall infections is the use of infected *Rhus chinensis* Mill. in biomedical applications, as it was found to have high antioxidant activities in infected leaf tissues, as well as antiviral, antibacterial, and antitumor function in the gall tissues [26, 27]. Therefore, the purpose of this study was to estimate the effects of gall infection on the contents of health-related compounds in the leaves of *L. chinense*. Our data show that the contents of polyphenol and the level of chlorogenic acid and rutin were increased in the infected leaves. Leaf extracts also exhibited higher antioxidant activities after infection. Our results indicate that infected leaves have potential use in pharmacological applications and may possibly be consumed as health food.

## Methods

### Plant material and sample collection

Goji (*L. chinense*) seeds were obtained from Miaoli District Agricultural Research and Extension Station (MDAIS) in Taiwan. Goji were planted in the MDAIS and the plant material was identified by Jhin-Syuan Wang in MDAIS. Plant specimen of local grown *L. chinense* is available in Herbarium of National Taiwan University (TAI, Link: https://tai2.ntu.edu.tw/specimen/specimen.php?taiid=206526). Normal uninfected and gall mite-infected Goji leaves were harvested in the winter, which is the appropriate season for growing Goji in Taiwan. Dried Goji berries (i.e. the fruit of Goji from *L. barbarum*) were purchased from the local market.

### Histology of gall tissues

Three different developmental stages (initiation, enlarging, and maturation) of gall tissues were collected and were fixed by formalin-acid-alcohol (FAA) fixative. After dehydration and paraffin wax infiltration, samples were sectioned using a tissue dissector (Leica RM2125 RTS, Leica, Germany) and were subsequently stained with Safranin O and Fast Green.

### Determination of chlorophylls and carotenoid

Dried samples were ground using liquid nitrogen, and the pigments were extracted with 80% acetone. After centrifugation, the supernatant was used for the measurement of absorbance at 663.6 nm, 646.6 nm, and 440.5 nm. Contents of chlorophyll and carotenoids were determined according to our previous report [28].

### Determination of flavonoids and polyphenols

The flavonoid content was determined according to Lesjak et al. [29]. A Range of 3.2 mg/mL – 8 mg/mL dried sample was ground for flavonoid extraction by using 90% methanol. After centrifugation, 30 μL of supernatant was diluted with 90 μL of methanol, following by adding 6 μL of 10% AlCl_3_ (Sigma-Aldrich) and 6 μL of 1 M sodium acetate (CH_3_COONa, Sigma-Aldrich). Absorbance was read at 415 nm after adding 170 μL of distilled water and a 30 min of waiting. Calibration curve was constructed by using 10 concentrations of quercetin (Sigma-Aldrich) ranging from 0.625 μg/mL to 320 μg/mL. *R*^2^ of calibration curve was 0.9983. Total flavonoid content was demonstrated as mg of quercetin equivalents (QE) per g of dried sample weight (DW).

Content of phenolic acids in the collected Goji samples were analyzed based on the method established by Singleton and Rossi [30]. Briefly, 90% methanol with 0.3% (v/v) HCl was added to 10 mg of powdered sample. After centrifugation, 100 μL of supernatant was mixed with 2 mL of 2% Na_2_CO_3_ solution and the absorbance at 750 nm was analyzed using spectrophotometry (Multiskan GO, Thermo Scientific). Gallic acid (Sigma) was used for plotting the calibration curve. The concentration of polyphenols was determined and presented as gallic acid equivalent (GAE).

### Determination of chlorogenic acid and rutin

The UPLC-MS system was using Agilent 1290 Infinity II ultra-performance liquid chromatography (UPLC) system (Agilent Technologies, Palo Alto, CA, USA) coupled online to the Dual AJS electrospray ionization (ESI) source of an Agilent 6545 quadrupole time-of-flight (Q-TOF) mass spectrometer (Agilent Technologies, Palo Alto, CA, USA). The sample was separated by using ACQUITY UPLC BEH C18 column (1.7 μm, 2.1 × 100 mm, Waters Corp., Milford, MA, USA). The column temperature was 35 °C. The mobile phase used for this study were double-distilled water (eluent A) and acetonitrile (eluent B). The gradient condition was: 0–2 min, 3% B; 2–9 min, 3–20% B; 9–17 min, 20–50% B; 17–25 min, 50–100% B; 25–27 min, 100% B; 27–28 min, 100–3% B, 28–30 min, 3% B. The flow rate was 400 μL/min and the injection volume of sample was 2 μL. The instrument was operated in negative full-scan mode and collected from an m/z of 100–1100. The MS operating conditions were optimized as follows: Vcap voltage, 3.0 kV; nozzle voltage, 1.0 kV; nebulizer, 30 psi; gas temperature, 250 oC; sheath gas temperature, 325 oC; sheath gas flow (nitrogen), 11 L/min; drying gas (nitrogen), 9 L/min. The chromatogram acquisition, detection of mass spectral peaks, and their waveform processing were performed using Agilent Qualitative Analysis 10.0 software (Agilent, USA). Quantitative determination was performed with reference to the calibration curve of internal chlorogenic acid and rutin standard. The R^2^ of calibration curves are 0.9941 of chlorogenic acid and 0.9968 of rutin.

### Determination of antioxidant capacity

The 1,1-diphenyl-2-picrylhydrazyl (DPPH) radical scavenging method that was previously reported [31] was modified as follows: 20 mg of sample powder was dissolved in 1 mL of 90% methanol prior to reaction with a methanolic solution of DPPH. Two hundred μL of extract/DPPH solution (1:3) were loaded to 96-well plate and the plate was placed in dark for 90 min for reaction. The absorbance at 517 nm was determined by spectrophotometer. The DPPH scavenging capacity of Goji extract was expressed as proportional (percentage) inhibition (% inhibition). The DPPH scavenging capacity of Goji extract was expressed as proportional (percentage) inhibition (% inhibition). The half maximal inhibitory concentration (IC50) of Goji extract on DPPH scavenging activity was calculated using a three parameter logistic regression model:

$$ \mathrm{Y}=\operatorname{Max}/\left(1+{\left(\frac{\mathrm{X}}{\mathrm{IC}50}\right)}^{\mathrm{Hill}\ \mathrm{coefficient}}\right) $$

### Statistical analysis

Statistics were assessed using the Student’s T test, and significant differences were presented as *p < 0.05, **p < 0.01, ***p < 0.001.

## Results

**Fig. 1:**
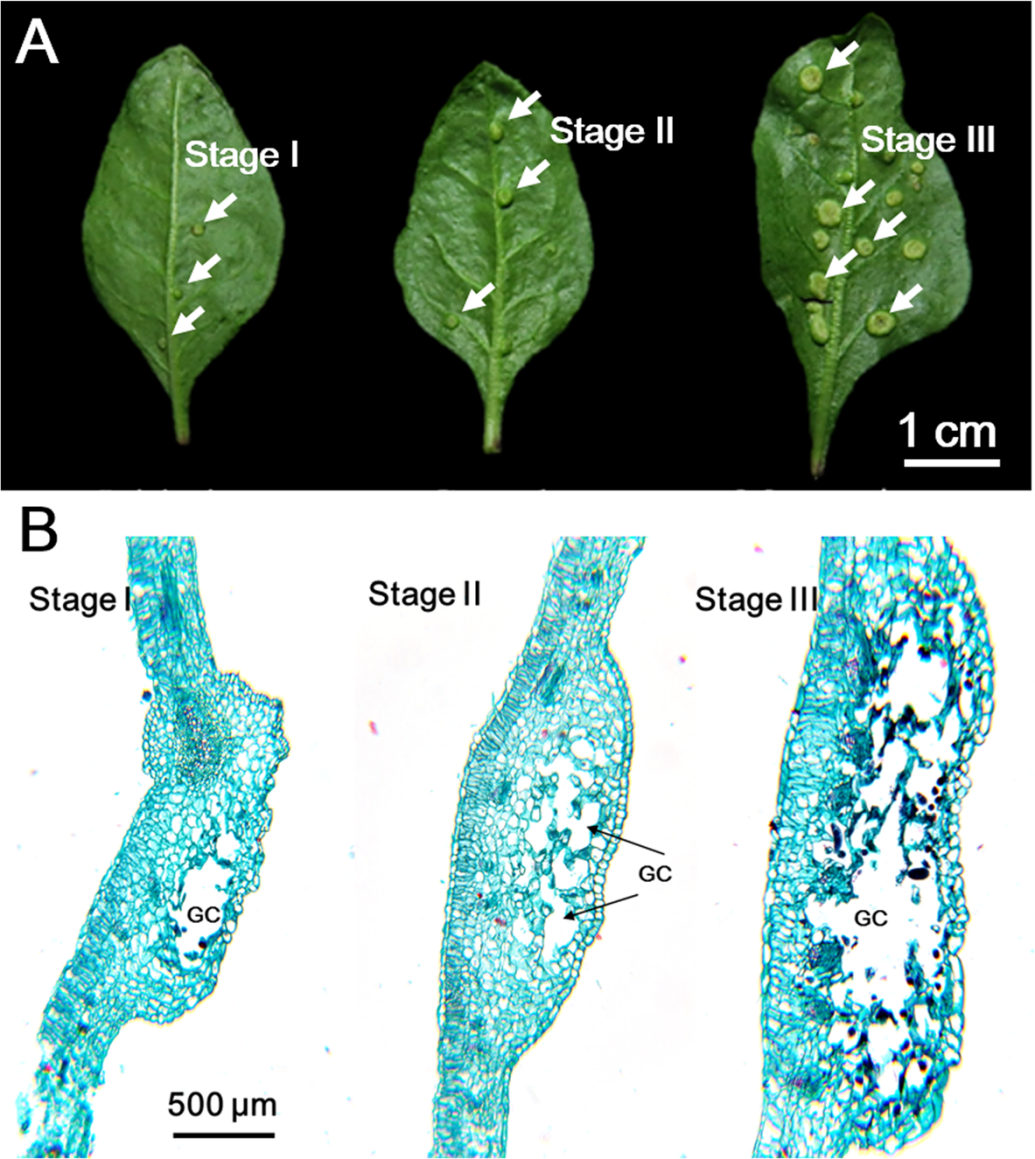
Morphology of gall tissue on the leaves of Goji. **a** Three developmental stages of gall tissues. **b** Section of gall tissue in three developmental stages. Arrow in (**a**), gall tissue. GC, gall chamber

**Fig. 2:**
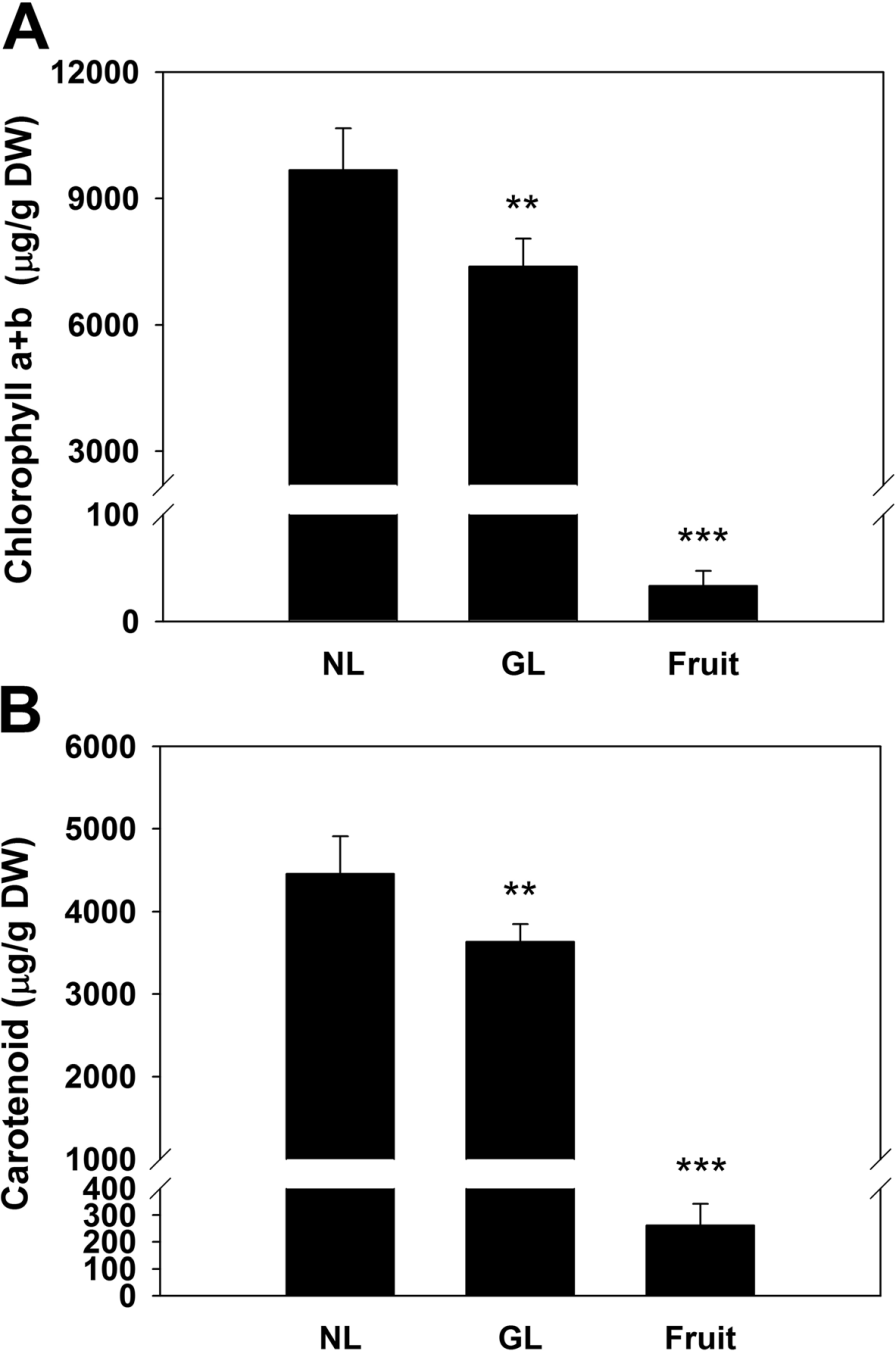
Content of Chlorophyll (**a**) and carotenoid (**b**) in the leaves and fruit. NL, normal uninfected leaves. GL, galled leaves. Fruit, Goji berry. Data are presented as mean ± S.D. (*n* = 6). *Significantly different against NL (Student’s *t*-test)

Figure 1 shows the three developmental stages of leaf galls. In early development (stage I), the gall chamber was small and was surrounded by nutritive parenchyma cells. The gall chamber was enlarged during development, but the number of parenchyma cells decreased (Fig. 1b). The analysis of photopigments showed that the contents of chlorophyll (chlorophyll a + b) and carotenoids were slightly decreased in galled leaves (Fig. 2).

**Fig. 3:**
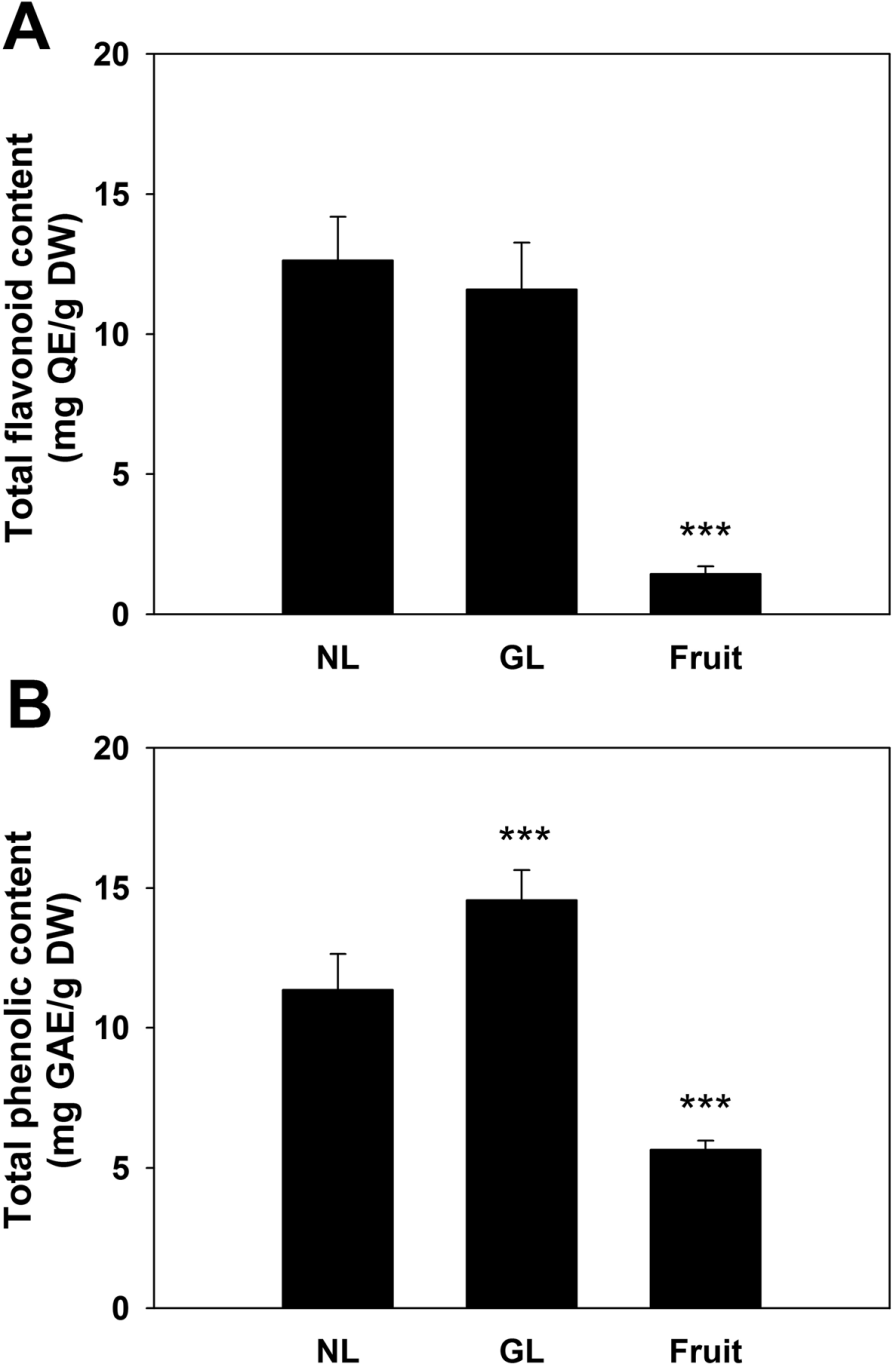
Content of flavonoids (**a**) and polyphenols (**b**) in the leaves and fruit. NL, normal uninfected leaves. GL, galled leaves. Fruit, Goji berry. Data are presented as mean ± S.D. (*n* = 6). *Significantly different against NL (Student’s *t*-test)

In plants, polyphenolic compounds and flavonoids are molecules that introduce defense-related mechanisms against pathogens and other types of stress. In this study, no significant change of flavonoids was shown in the galled leaves (Fig. 3a). The polyphenol content was higher in galled leaves, showing an elevation of content by ~ 30% (Fig. 3b). The content of polyphenols and flavonoids were relatively lower in the Goji berry.

**Fig. 4:**
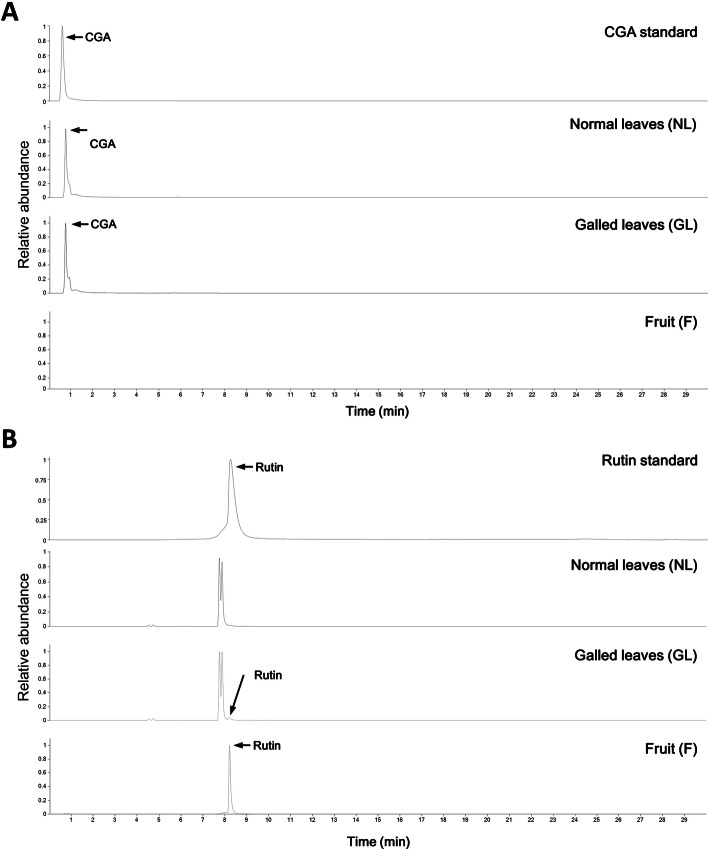
Extracted-ion chromatograms (EICs) of standards and Goji extracts in the negative ion mode. **a** Plotting EICs of chlorogenic acid (CGA) signal. **b** Plotting EICs of rutin signal. The split peak occurs in NL and GL was considered to be a rutin isomer

The content of chlorogenic acid, which is one of the main polyphenolic compounds in Goji leaves, in each sample were detected by UPLC-MS/MS and the MS chromatogram was showed in Additional Figure 1. Chromatograms of identified chlorogenic acid and rutin in NL, GL, and fruit were showed in Fig. 4. The average level of chlorogenic acid in infected leaves was ~ 36% higher than that in non-infected leaves (Table 1). Table 1 also reveals the content of rutin in galled leaves and Goji berry. The content of rutin in normal leaves, however, was beyond the lower limit of detection.

**Table 1:** Contents of chlorogenic acid (CGA) and rutin in 3 types of Goji extracts

Compounds	Normal leaves	Galled leaves	Fruit
Chlorogenic acid	5004.7 ± 698.6	6815.6 ± 1389.1*	–
Rutin	–	53.6 ± 37.5	439.3 ± 212.2

Average content was presented as mean ± S.D. (*n* = 5–6)

* *p* < 0.05 (Student’s t-test, comparing to normal leaves)

**Fig. 5:**
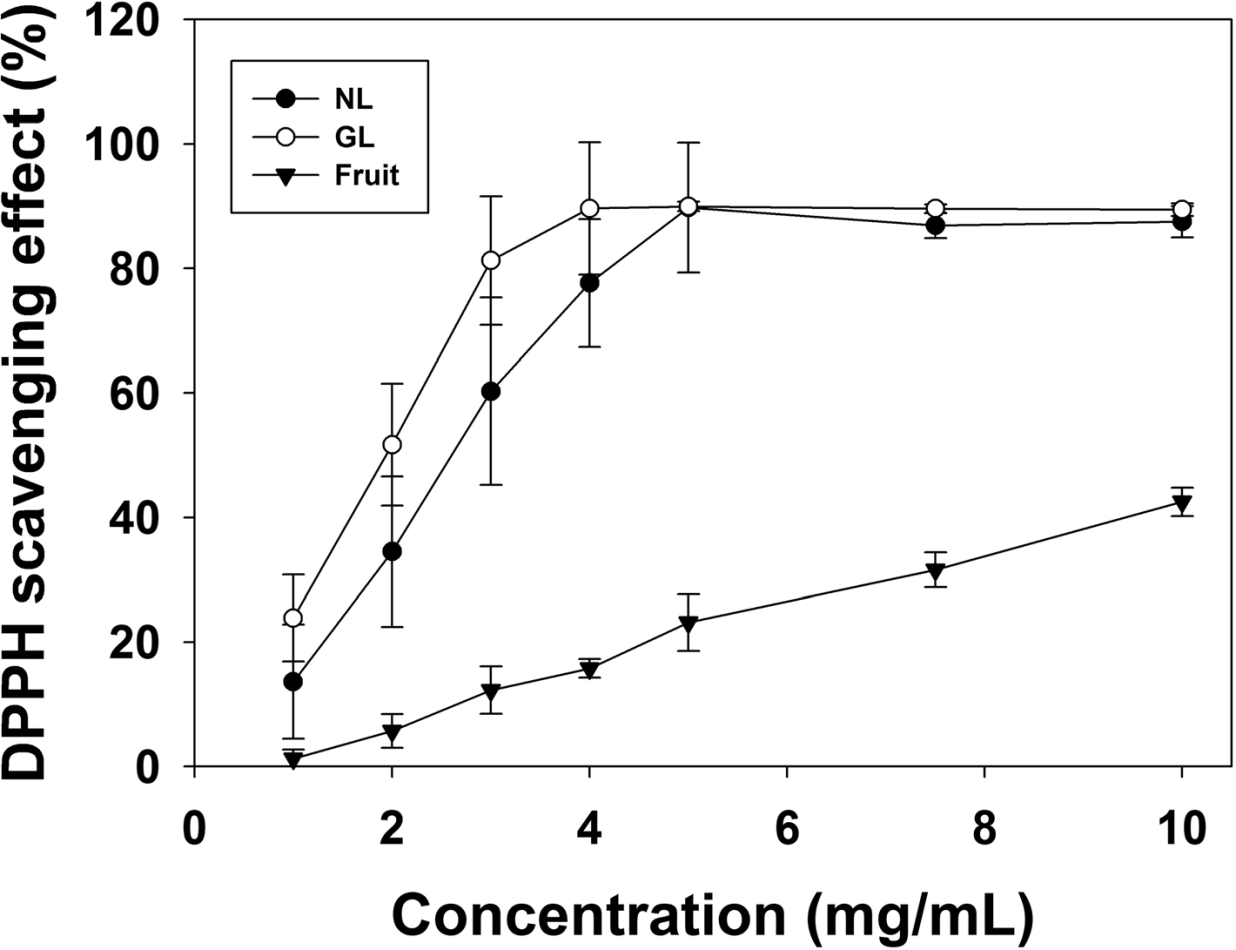
Antioxidant capacity of Goji extracts. Inhibitory effects of different concentrations of Goji extracts on scavenging DPPH. NL, normal uninfected leaves. GL, galled leaves. Fruit, Goji berry. Data are presented as mean ± S.D. (*n* = 6)

**Fig. 6:**
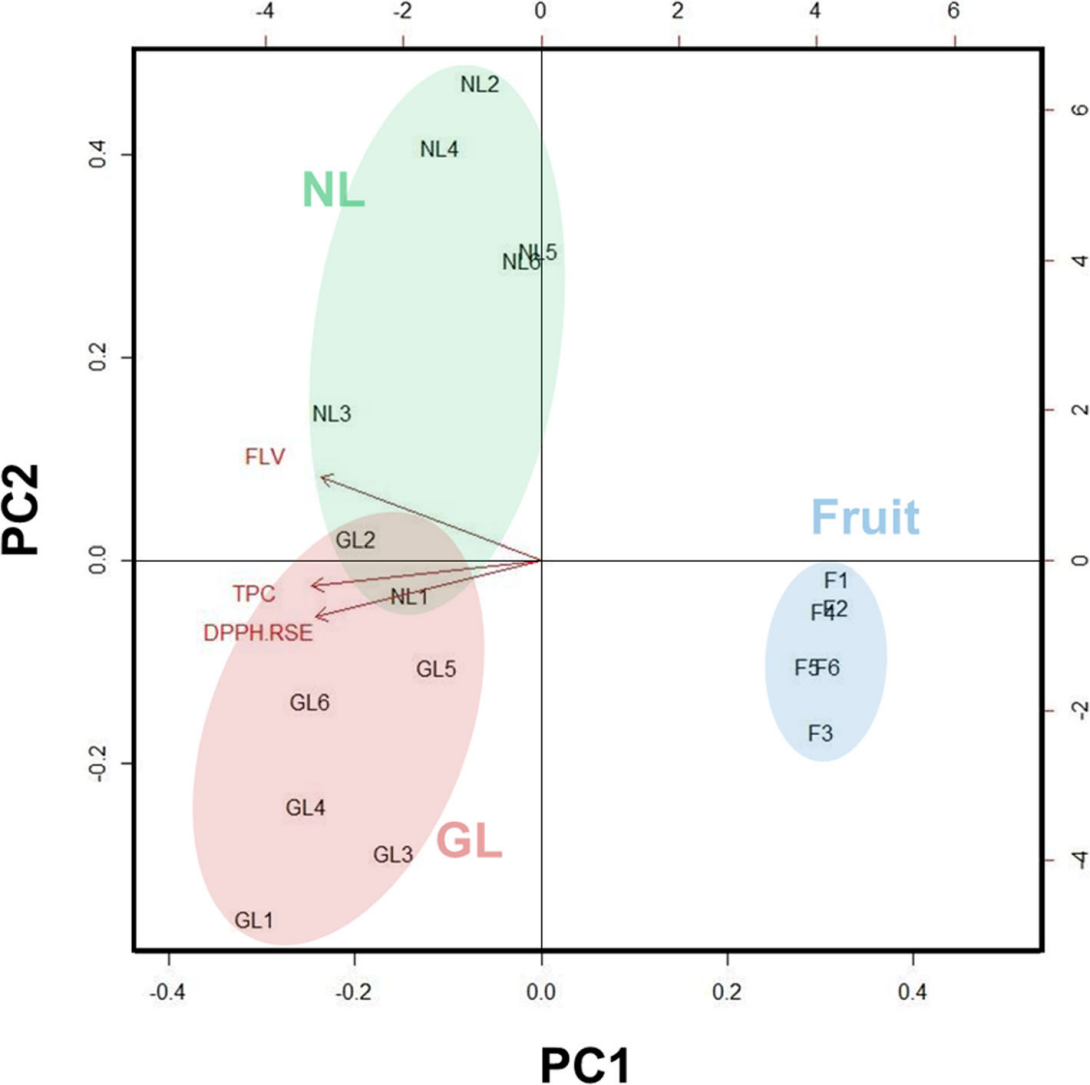
Principal component analysis (PCA) biplot of the flavonoids (FLV), total phenolic content (TPC), and the antioxidant activity in Goji extracts. Six replicates in each Goji extract, total 18 loadings were analyzed. Antioxidant activity was loaded as the DPPH radical scavenging effect (DPPH RSE, in %) of 2 mg/mL in each extract. NL, normal uninfected leaves. GL, galled leaves. Fruit, Goji berry

Antioxidant activity of the extracts from the Goji leaves and fruit are presented in Fig. 5. Results show that infected leaves contain higher DPPH scavenging ability than that of normal leaves (Fig. 5, Table 2). The extracts of Goji berry, on the other hand, showed the lowest ability in scavenging DPPH and among the three samples analyzed. Principal component analysis (PCA) showed the relation of the amount of flavonoids (FLV), polyphenols and antioxidant ability (DPPH radical scavenging effect) in Goji extracts (Fig. 6).

**Table 2:** IC50 (mg/mL) of DPPH scavenging ability of Goji leaves and fruit

	Normal Leaves	Galled Leaves	Fruit	BHT
IC50	2.41 ± 0.40	1.65 ± 0.34*	11.49 ± 0.76***	0.15 ± 0.02

IC50 was presented as the average value of 6 activity curves in each sample

*, *p* < 0.05; ***, *p* < 0.001 (Student’s *t*-test, comparing to normal leaves)

## Discussion

Loss of photosynthesis ability and associate pigments and proteins is a main change during leaf gall formation. The lack of photosynthesis-related pigments and protein complexes in gall tissues on leaves have been demonstrated in previous studies and was recognized as a shift from autotrophy to heterotrophy [32, 33]. Commercial dried Goji fruit were used for comparing the constituents in the leaves and in the Goji berry that people usually consume. The data showed that Goji berry contains a relatively low amount of both chlorophyll and carotenoids. The carotenoid content of Goji berry is comparable to that of the previous report [34]. The leaves contained more than a tenfold higher total carotenoid levels, suggesting higher antioxidant capability in the leaves. Notably, Goji berry are considered to be a source of macular pigments [4], it would be worthy to test if Goji leaves is able to contribute more since it contain a higher amount of carotenoid.

The higher antioxidant ability may be provided by the increased contents of secondary metabolites. Reports done by our group, as well as other researchers, have demonstrated that infection of the gall usually elevates the contents of phenolic acids and flavonoids in gall tissue [25, 35]. In this study, we found that the polyphenol content was higher in the infected leaf (Fig. 3b) Contents of flavonoids and polyphenols in the leaves have been previously reported in the study of methanol or ethanol extracts from *L. barbarum* and *L. chinense* [16, 17, 36]. Higher phenolic acids in leaves could be found in cultivated *L. barbarum* compared to that in wild *L. barbaraum* [17]. Content of polyphenols in leaf extracts is comparable to the previous report which used methanol for extraction as well [17], but is much lower than that extracted by ethanol [16]. Moreover, reports have also shown higher amounts of polyphenols and flavonoids in the leaves of *L. chinense* than in *L. barbarum* [16]. Accordingly, a higher amount of phenolic constituents could be obtained from the galled leaves of particular cultivars of *L. chinense*, as the value of polyphenols in leaves from this study had doubled after infection (Fig. 3).

Chlorogenic acid is one of the main compounds found in Goji leaves [15, 16, 36]. Comparing the polyphenols of extracts from the leaves of *L. chinense* to those in *L. barbarum*, the amounts of chlorogenic acid is twofold higher [16]. In this study, we compared the galled and ungalled leaves of *L. chinense* and showed that the average level of chlorogenic acid was increased after gall induction. Both our report and previous reports have demonstrated higher chlorogenic acid content in leaves than in Goji berry [18], as seen in Table 1 of the present study. According to our research, galled Goji leaves have potential in contributing more of the protective effects against oxidative stress, inflammation, and other hepato- and cardio-related syndromes [37, 38]. In this study we also showed that the level of rutin was increased after gall induction. Our data have slight differences from a recent report which showed that the rutin content in fruits is lower than that in leaves of *L. barbarum* [18]. This inconsistency might be caused by the difference in the Goji cultivar analyzed.

After testing the antioxidant activity of the extracts from the Goji leaves (Fig. 5, Table 2), our data showed that the antioxidant capacity of leaf extracts was elevated after gall formation. PCA result demonstrate that three variables (content of flavonoid and polyphenols, and DPPH scavenging effect) are positive correlated (Fig. 6). The smaller angle formed between vectors of polyphenol content and DPPH scavenging effect indicate a higher correlation of phenolic acids and antioxidant ability. PCA analysis also presents the higher contribution of galled leaves in radical scavenging (Fig. 6). In many other plant species, including Ficus and Aleppo oak, gall tissues also have higher antioxidant activity according to the DPPH assays presented in other studies [39, 40]. The increase of antioxidant activity in gall tissue, which partially results from the elevated phenolic compound content, is a defense response of the plant against parasites. It was also suggested that the senescence of gall tissue is inhibited as the level of antioxidants remains high [41].

## Conclusions

Goji leaves are currently considered as a health food. Because the galled Goji leaves have elevated levels of phenolic acids and possess stronger antioxidant ability, it should be considered as a health food instead of being regarded as waste. The elevation of chlorogenic acid and rutin level after infection indicates a higher pharmaceutical or nutraceutical value the galled leaves have. An examination of the effect of gall infection on the Lycium cultivar(s) enriching chlorogenic acid is worthy for determining the application foreground of Goji leaves. The Goji leaves also being a good choice as alternative to Goji berry for the people preferring to assimilate less sugar and have more dietary fiber. In addition, promoting the non-use of pesticides in controlling gallers will have environmental benefits. However, our study found that in Goji leaves, the level of salicylic acid, a plant defense-related compound that has been suggested to be allergenic, is elevated after infection (data not shown). A nutraceutical food should be certain in the case that galls do not affect human health. Therefore, the pharmaceutical value of infected Goji leaves also need to be carefully evaluated.

## Supplementary information

**Additional file 1: Figure S1.** Total ion chromatograms of Goji extracts. NL, normal leaves; GL, galled leaves; F, fruit.

**Additional file 2: Figure S2**. Fragmentation of chlorogenic acid and rutin standards in MS spectra.

## Data Availability

All raw data and specimen are available at the Department of Horticulture and Landscape Architecture in National Taiwan University, contact person Yu-Sen Chang.

## Abbreviations

CGA: Chlorogenic acid
DPPH: 1,1-diphenyl-2-picrylhydrazyl
EDTA: Ethylenediaminetetraacetic acid
GAE: Gallic acid equivalent
MRM: Multiple Reaction Monitoring
PCA: Principle component analysis
QE: Quercetin equivalent
S.D.: Standard deviation
UPLC: Ultra Performance Liquid Chromatography
