# Supplementary figures and images for: Potential of galled leaves of Goji (Lycium chinense) as functional food
Source: BMC Nutr. 2020 Jul 7;6:26. doi: 10.1186/s40795-020-00351-w (PMC7339520; doi:10.1186/s40795-020-00351-w)

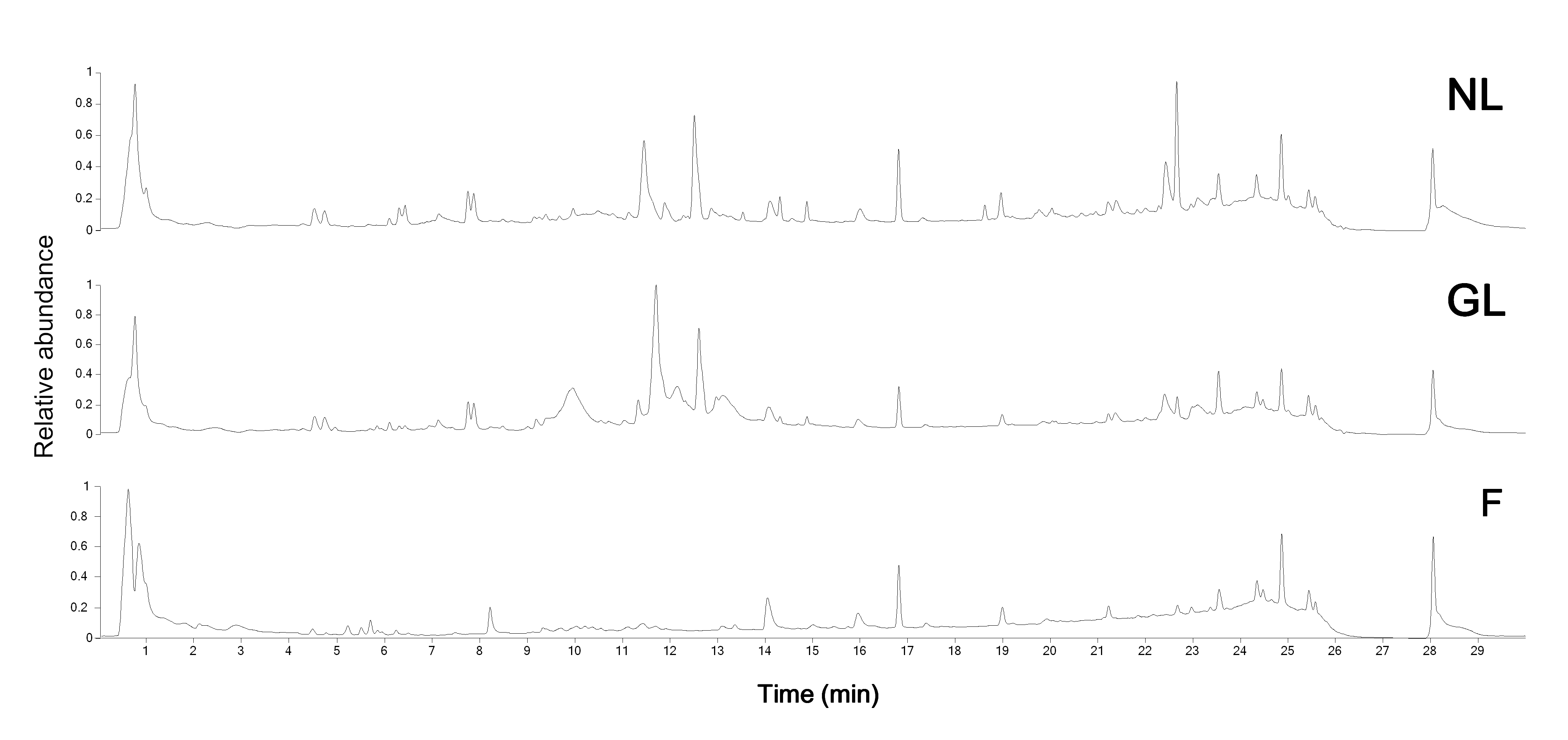

Supplement: Supplementary file 1 — Additional file 1: Figure S1. Total ion chromatograms of Goji extracts. NL, normal leaves; GL, galled leaves; F, fruit. [file 40795_2020_351_MOESM1_ESM.jpg]

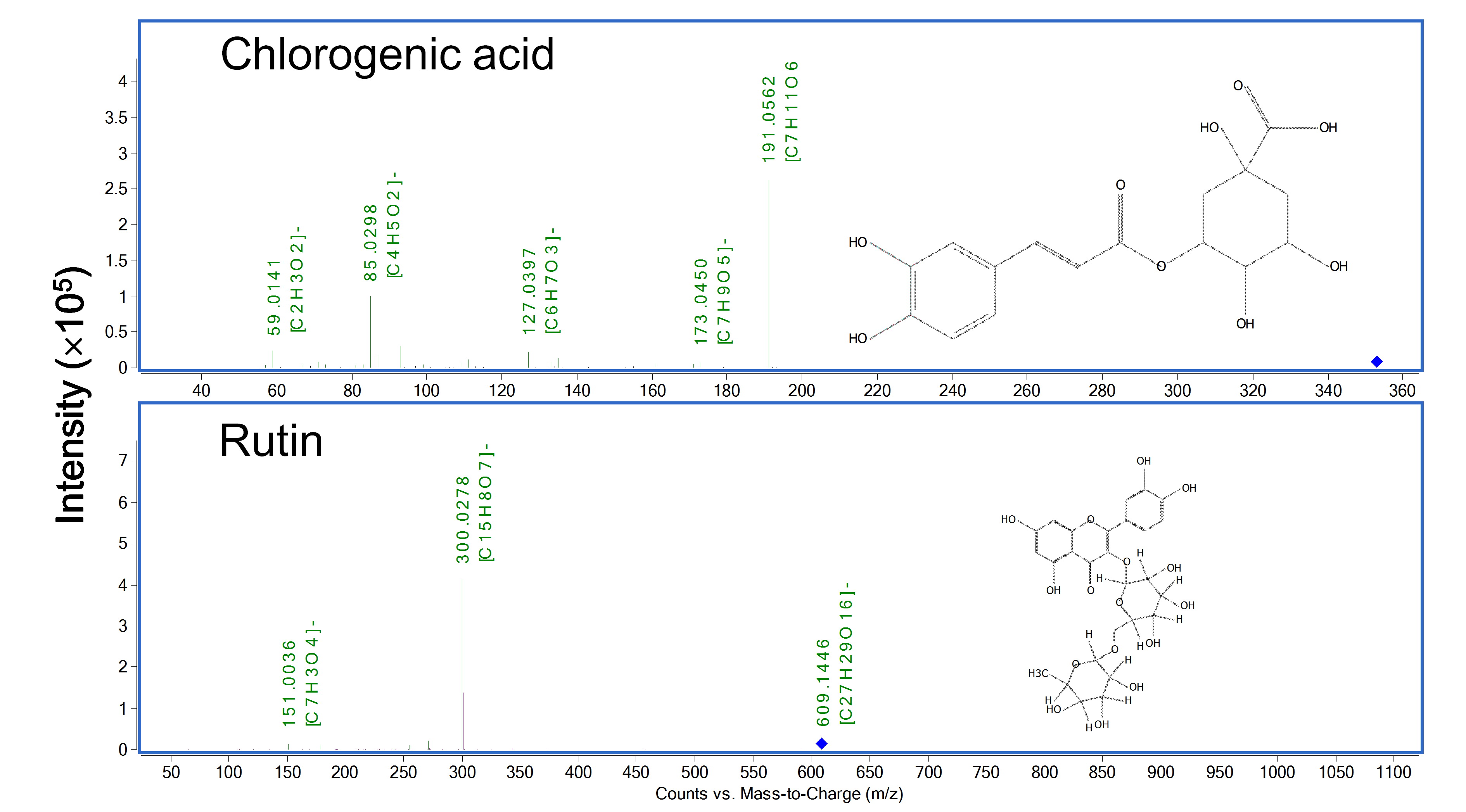

Supplement: Supplementary file 2 — Additional file 2: Figure S2. Fragmentation of chlorogenic acid and rutin standards in MS spectra. [file 40795_2020_351_MOESM2_ESM.jpg]
